# Mental health providers are inexperienced but interested in telehealth-based virtual reality therapy: survey study

**DOI:** 10.3389/frvir.2024.1332874

**Published:** 2024-07-03

**Authors:** Triton Ong, Janelle F. Barrera, Charvi Sunkara, Hiral Soni, Julia Ivanova, Mollie R. Cummins, Kaitlyn R. Schuler, Hattie Wilczewski, Brandon M. Welch, Brian E. Bunnell

**Affiliations:** 1 Doxy.me Research, Doxy.me Inc., Rochester, NY, United States; 2 Department of Psychiatry and Behavioral Neurosciences, Morsani College of Medicine, University of South Florida, Tampa, FL, United States; 3 Lake Erie College of Osteopathic Medicine, Bradenton, FL, United States; 4 College of Nursing and Department of Biomedical Informatics, University of Utah, Salt Lake City, UT, United States; 5 Biomedical Informatics Center, Medical University of South Carolina, Public Health and Sciences, Charleston, SC, United States

**Keywords:** virtual reality, telehealth, mental health therapy, telemental health, tele-VR

## Abstract

Virtual reality (VR) is an emerging technology that can enhance experiences and outcomes in mental healthcare. However, mental health therapists have been slow to adopt VR into practice. Implementation of telehealth-based VR therapy (tele-VR) could catalyze adoption and innovation in mental healthcare. To explore therapists’ perspectives on tele-VR, we conducted a cross-sectional survey of practicing mental health providers in the United States in June-July 2023. We analyzed 176 completed surveys from therapists, of whom 51.14% had no prior experience with VR, only 6.25% had used VR clinically, and 56.82% had neutral impressions of VR for therapy. Despite therapists’ general inexperience with VR, therapists indicated a wide variety of tele-VR simulations (e.g., social situations, flying, heights) and features (e.g., personalized spaces, homework, interactivity) would be moderately to extremely useful for their practices. Therapists also requested additional VR simulations and features for their telehealth clients such as behavioral skills training, exposure therapy, gender identity therapy, and psychological assessments in VR. Therapists rated Health Insurance Portability and Accountability Act compliance, the ability to try VR before buying, affordability for therapists, accessibility for clients, and insurance coverage as the five most influential implementation factors for tele-VR. Overall, therapists were generally inexperienced and neutral about VR for telehealth therapy, but were interested in tele-VR for specific applications. These findings provide actionable directions for future research and collaborative development of therapeutic VR content and features.

## 1 Introduction

Mental health providers need innovative solutions to meet growing and global demand for therapy (Patel et al., 2023). Telehealth and virtual reality (VR) are promising technologies that can improve access to and provision of mental healthcare. Telehealth, the use of telecommunications technologies such as the internet to deliver synchronous or asynchronous health services (Doraiswamy et al., 2020), has become well established in mental health fields (Zangani et al., 2022). VR, which uses interconnected sensors and encompassing displays to provide highly immersive simulated experiences (Snoswell and Snoswell, 2019), has also been demonstrated to make therapy more approachable, enjoyable, and impactful for people with anxiety, post-traumatic stress disorder (PTSD), obsessive-compulsive disorder (OCD), eating disorders, and depression (Dellazizzo et al., 2020; Albakri et al., 2022; Ciążyńska and Maciaszek, 2022; van Loenen et al., 2022; Li et al., 2023). However, while mental healthcare has become the most common use of telehealth in recent years (Trilliant Health, 2022), most mental health therapists have yet to deploy VR in their services.

Mental health providers have reported a variety of perceived barriers to using VR clinically. While therapists no longer viewed costs as a leading barrier (Lindner et al., 2019a), they reported a lack of training and difficulty finding VR content designed specifically for clinical mental healthcare (Rizzo and Koenig, 2017; Boeldt et al., 2019; Cieślik et al., 2020; Pimentel et al., 2021; Wray et al., 2023). Therapists also described perceptions that clients would be skeptical about VR therapy and its potential side effects (Chung et al., 2023). Interestingly, these perceived barriers may not reflect reality. In a 2021 study of therapists who used VR in practice, the therapists agreed universally that VR was a valuable tool (100%), they would recommend VR to fellow mental health professionals (100%), clients had positive reactions to VR therapy (100%), and believed VR helped clients in ways not possible via alternative approaches (93.8%) (Vincent et al., 2021). Despite promising clinical findings, enthusiastic support among therapist adopters, and growing consumer interest, recent surveys found only 0.1%–13% of mental health providers have used VR in their practice (Lindner et al., 2019a; Sampaio et al., 2021; Chung et al., 2022; Preston et al., 2022).

It is vital to explore the gaps between research and practice to understand paths towards adoption of VR for mental healthcare. Telehealth may be an important way to improve the way therapists provide VR therapy to their clients. Telehealth is used for mental healthcare across the globe (Zangani et al., 2022), with clinical outcomes and satisfaction comparable to those of in-person care (Batastini et al., 2021; Mazziotti and Rutigliano, 2021; Giovanetti et al., 2022; Lin et al., 2024). Therapists and clients have embraced the ease, efficiency, efficacy, effectiveness, and convenience of telehealth for mental healthcare (Batastini et al., 2021; Butzner and Cuffee, 2021; Siegel et al., 2021; Steidtmann et al., 2022; Connolly et al., 2024). Similarly, there is good reason to expect Clients’ preferences for convenient care and therapists’ concerns about feasibility can both be addressed by VR therapy delivered via telehealth (i.e., tele-VR) (Di Carlo et al., 2021; Sampaio et al., 2021). For example, an online VR platform reduced shyness and improved self-esteem for people with quadriplegia engaging in a therapeutic group singing intervention (Tamplin et al., 2020). Researchers in another study demonstrated synchronous VR-based cognitive behavioral therapy that helped therapists build trust rapidly with young women at risk for eating disorders (Matsangidou et al., 2022); however, while therapists and participants never met in-person, participants still needed to travel to the study site in order to access the VR equipment. While research has demonstrated VR-based mental health interventions conducted in participants’ homes (Lindner et al., 2019b; Shin et al., 2021; Worlikar et al., 2023), the vast majority of these applications were self-guided and did not involve live interaction with a therapist in VR. The potential of synchronous tele-VR mental health therapy remains understudied.

Tele-VR presents unprecedented opportunities to improve engagement and personalization of mental healthcare experiences (Ong et al., 2022). While exciting, tele-VR is a new area of research that has become possible only recently. The proliferation of portable, affordable, and popular consumer VR devices has enabled people to access new forms of telehealth-based VR therapy from the comfort of their own homes. In a recent example, researchers provided Meta Quest 2 VR headsets to children with disabilities and played social VR video games to alleviate their depression and loneliness (Lai et al., 2023). Previous studies have described wide varieties of mental health-related VR content (Arnfred et al., 2023; Sunkara et al., 2023), discussed the clinical potential of VR features (Valmaggia et al., 2016; Boeldt et al., 2019), and identified therapists’ perceived barriers to adoption of in-person VR (Chung et al., 2021; Chung et al., 2022; Chung et al., 2023). However, to our knowledge, no studies have focused on content, features, and implementation of VR therapy delivered over telehealth. As the gatekeepers of mental healthcare, therapists’ perspectives are particularly important to inform the design, development, and implementation of tele-VR solutions. The purpose of this study was to explore telemental health providers’ perspectives of VR therapy and how they prioritize tele-VR simulations, features, and implementation factors for their clinical practices.

## 2 Methods

### 2.1 Study design

We conducted a cross-sectional survey of telemental health providers in the United States.

### 2.2 Participant recruitment

We recruited a convenience sample of practicing mental health therapists via TelehealthEngage. TelehealthEngage is a research registry of more than 5,000 healthcare professionals across a variety of specialties on the Doxy.me telemedicine platform, approximately 40% of whom specialized in mental healthcare. We notified members they would receive a $75 eGift card for completing the survey and invited them to participate if they were actively practicing mental healthcare in the United States, spoke English fluently, were at least 18 years old, and had a Master’s or Doctoral degree at the time of the study.

### 2.3 Survey design and procedures

We administered the survey from June 15 to July 3 of 2023 using Qualtrics with categorical, Likert-scale, and text response items across 7 sections (Supplementary Appendix S1). We presented tele-VR simulations, features, and implementation factors derived from previous research on therapist perspectives on VR therapy (Chung et al., 2021; Chung et al., 2022; Arnfred et al., 2023; Chung et al., 2023; Sunkara et al., 2023; Ong et al., 2024).

#### 2.3.1 Screening and informed consent

Participants began the survey after accepting the email invitation. The initial 5 questions screened for age, English fluency, degree, mental healthcare specialty, and telehealth caseload. Participants who passed the screening questions then completed an electronic informed consent form before accessing the rest of the survey. If a participant failed to meet screening criteria or declined the informed consent, we thanked them for their interest and dismissed them from the survey.

#### 2.3.2 Personal and professional demographics

We asked participants 3 questions about their personal demographics (i.e., gender, ethnicity, and race) and six questions about their professional characteristics (i.e., years practicing mental healthcare, years using telehealth, type of clinical organization, primary source of reimbursement, primary client age group, and up to three primary mental health disorders treated). If a participant selected Other as a primary mental health disorder in their therapy, we then asked 1 optional open-ended question to describe their clinical specialty.

#### 2.3.3 VR background

We presented up to 3 questions to explore participants’ backgrounds with VR. To explore participants’ prior experience with VR generally (i.e., not limited to clinical use), we asked 1 question on a 5-point Likert scale (1 = *No Experience* to 5 = *Extremely Experienced*). If a participant’s experience was any greater than *No Experience*, we asked 1 question about how often they used VR for therapy (5-point Likert scale from 1 = *Never* to 5 = *Frequently*). Then, we asked all participants 1 question about their overall impression of VR (5-point Likert scale from 1 = *Very Negative* to 5 = *Very Positive*).

#### 2.3.4 Overview video

**FIGURE 1:**
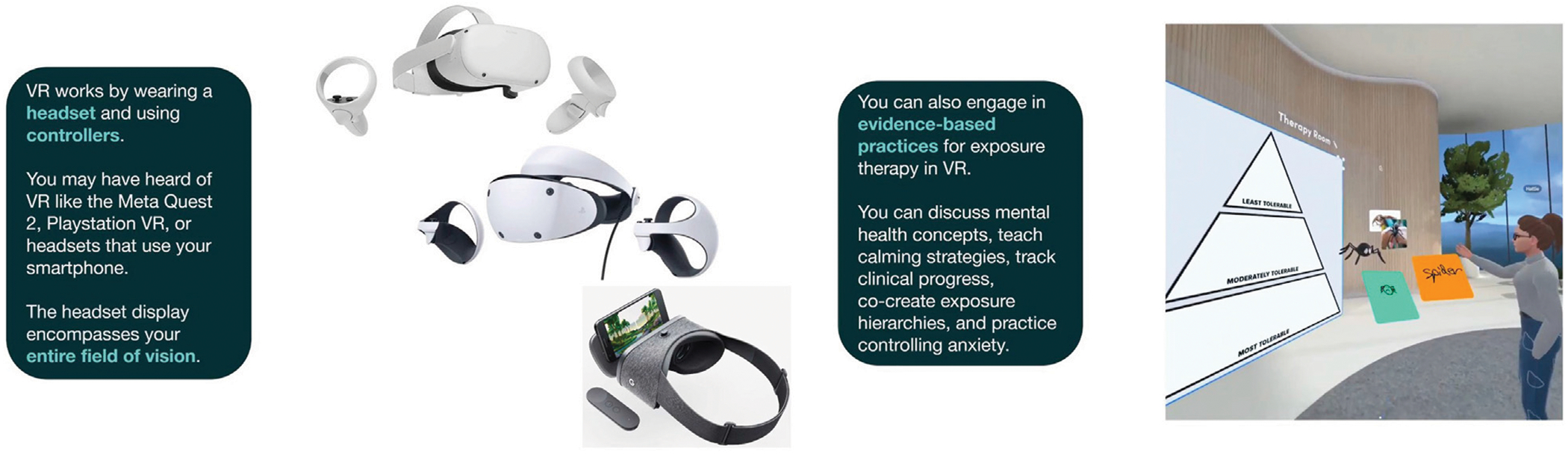
Video screenshots. Common VR headsets (left) and depiction of collaborative exposure hierarchy creation in tele-VR (right).

We produced a 4-min, 20-s video to familiarize participants with VR, telehealth, and tele-VR prior to asking the remainder of the survey on these topics. The video included voiced, textual, and visual depictions of typical VR hardware (e.g., all-in-one headset with handheld controllers), VR software features (e.g., immersive and multiuser VR), clinical evidence supporting VR for mental health therapy, and how tele-VR therapy might work in practice (i.e., remote, synchronous, avatar-mediated, immersive conversation and interaction with 3D therapeutic content). The video included a detailed example of how a therapist and client might use tele-VR to conduct exposure therapy for arachnophobia. Participants were required to watch the video for its entire duration before progressing to the next section (Figure 1).

#### 2.3.5 Tele-VR simulations

After viewing the tele-VR video, we asked participants to rate the usefulness of 12 tele-VR simulations on a 5-point Likert scale (1 = *Not Useful at All* to 5 = *Extremely Useful*), based on previous research (Arnfred et al., 2023; Sunkara et al., 2023; Ong et al., 2024). The simulations included VR objects or situations such as driving a car, small animals, and serious accidents, among others (Supplementary Appendix S1).

#### 2.3.6 Tele-VR features

We asked participants to rate the usefulness of 6 tele-VR features on a 5-point Likert scale (1 = *Not Useful at all* to 5 = *Extremely Useful*), based on previous research (Valmaggia et al., 2016; Boeldt et al., 2019; Ong et al., 2024). Features included tasks, activities, or actions to facilitate VR therapy such as personalizing therapeutic spaces, immersive interactions, tele-VR mental health exercises for clients to complete on their own, and others (Supplementary Appendix S1). We also asked 1 optional open-ended question for participants to describe other potentially useful tele-VR simulations or features.

#### 2.3.7 Tele-VR factors

We asked participants to rate the influence of 16 tele-VR implementation factors on a 5-point Likert scale (1 = *Not At All Influential* to 5 = *Extremely Influential*), based on previous research (Chung et al., 2021; Chung et al., 2022; Chung et al., 2023; Ong et al., 2024). Factors included statements such as, VR is accessible to my telehealth clients regardless of their age, sex, race, or other socioeconomic factors; VR therapy is secure, private, and in compliance with policies such as HIPAA or GDPR; VR attracts new clients to my telehealth practice; and others (Supplementary Appendix S1).

Upon completion of all survey sections, we asked participants to provide an email address to which we would send the $75 eGift card. Submitting an email address concluded the survey with a message of thanks to confirm participation.

### 2.4 Data analysis

We performed analyses using JASP (version 0.17.3) and Microsoft Excel 365 (version 2307). Participants’ data were excluded from analysis if they completed less than 100% of the required survey items. Primary data analysis consisted of descriptive statistics and frequencies.

We used Excel to qualitatively analyze responses to the one optional, open-ended question about suggestions for additional tele-VR simulations and features. First, we excluded irrelevant responses such as, “N/A” or “not that I can think of.” We segmented remaining responses into discrete suggestions if a participant provided multiple features or simulations in their response. We then read through discrete responses and grouped suggestions together based on mental health therapy context.

## 3 Results

### 3.1 Participant characteristics

We invited 897 potential participants, 218 of whom initiated the survey. However, 3 participants did not have a Master’s or PhD degree, 1 was not actively practicing telemental health at the time of the study, and 38 abandoned the survey. We analyzed the remaining 176 completed surveys (Table 1), which required about 15 min for participants to complete (*M* = 15.86 min, *SD* = 9).

Participants were generally middle-aged adults (*M* = 50.3 years, *SD* = 12.5, range 25–79), female (75.6%), non-hispanic (93.2%), and white (84%). Participants most commonly had a Master’s degree (56.3%), were psychologists (38%) or mental health counselors (27.3%), practiced as a solo provider (72.7%), took private insurance (64.8%), used telehealth for all of their clients (40.3%), and treated adult clients (84.1%). Participants had been practicing mental health for 18.9 years on average (*SD* = 9.8, range 2–45) and had been using telehealth for 3.8 years on average (*SD* = 1.6, range 1–10, mode = 3). The three most commonly treated mental health disorders were anxiety (87.4%), depression (79.9%), and trauma and stress-related disorders (63.5%). Of the 9 therapists who selected Other as a primary disorder in their mental health practice, 5 described their clinical focus to include gay men’s mental health (*n* = 1), individual outpatient psychotherapy (*n* = 2), and relational challenges (*n* = 2).

### 3.2 VR background

We asked participants about their experience with VR in general, how often they had used VR for therapy, and their overall impression of VR (Table 2). About half of participants had no experience with VR (51.1%), while the others were slightly (27.3%), somewhat (10.2%), moderately (6.8%), or extremely experienced (4.5%). Of the 86 participants who had at least some experience with VR, most had never used VR for therapy (42.6%) or used it for therapy rarely (0.6%), sometimes (2.3%), or frequently (3.4%). We then asked all participants about their overall impression of VR, which was mostly neutral (56.8%), somewhat positive (21%), or very positive (10.2%) with others either somewhat negative (8.5%) or very negative (3.4%).

### 3.3 Tele-VR simulations

**FIGURE 2:**
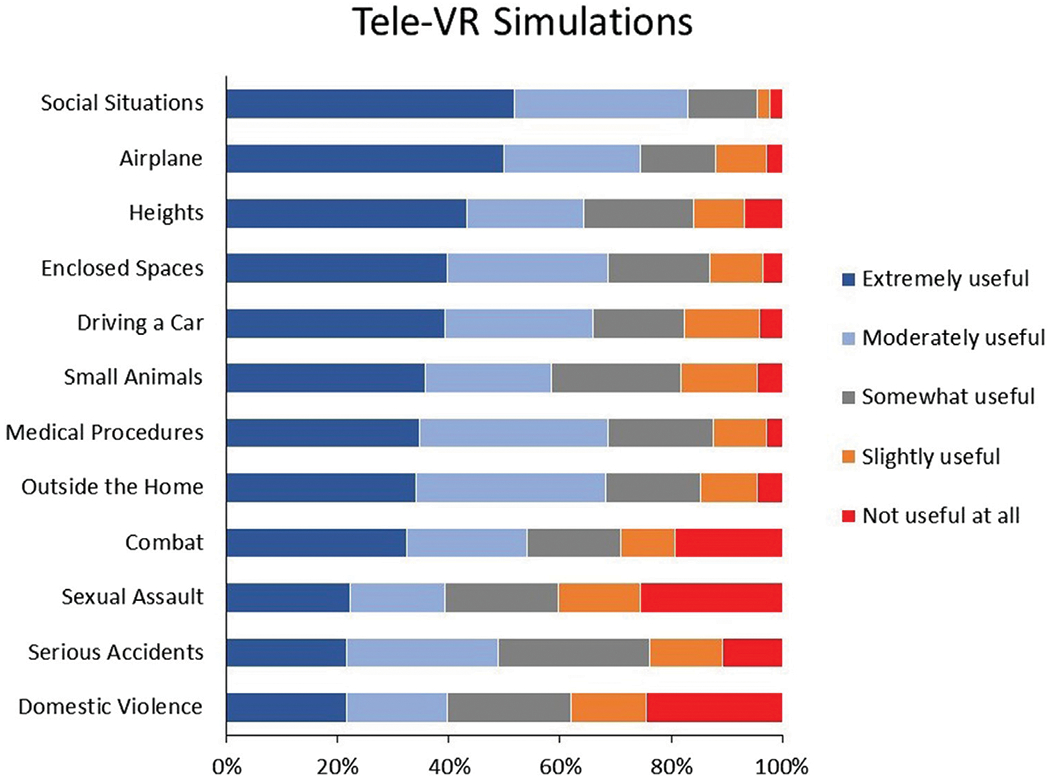
Therapist ratings of Tele-VR simulations.

Therapists rated their perceived usefulness of various tele-VR simulations on a 5-point Likert scale from *Not Useful At All* to *Extremely Useful* (Figure 2). Social situations was the highest-rated VR simulation (83% rated *Moderately* or *Extremely Useful*), followed by flying on an airplane (74.4%), enclosed spaces (68.8%), medical procedures (68.8%), and errands outside the home (68.2%). Driving a car (65.9%), heights (64.2%), and small animals (58.5%) were also rated with favorable usefulness. Combat (54%), serious accidents (48.9%), domestic violence (39.8%), and sexual assault (39.2%) were rated as the least useful VR simulations.

### 3.4 Tele-VR features

**FIGURE 3:**
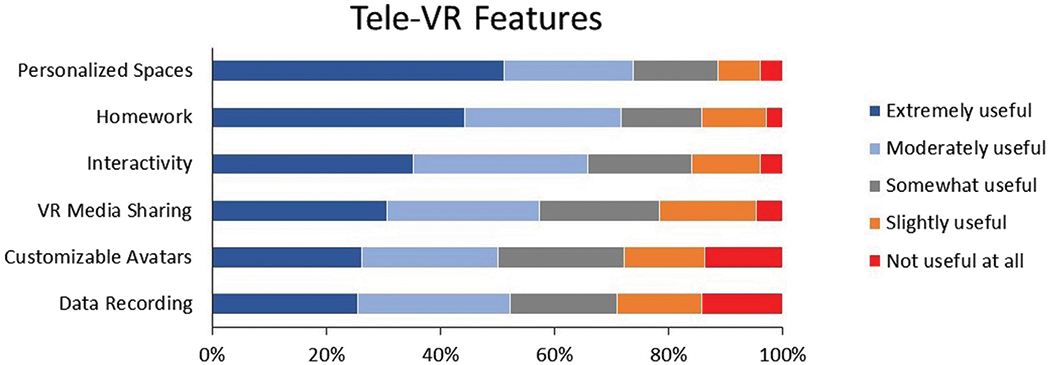
Therapist ratings of Tele-VR features.

Participants rated tele-VR features on a 5-point Likert scale from *Not Useful At All* to *Extremely Useful* (Figure 3). Personalizing therapeutic spaces (73.9%) was rated as the most useful VR feature, followed by VR homework (71.6%). Immersive activities (65.9%) and VR media sharing (57.4%) were also rated as favorably useful (i.e., either *Extremely Useful* or *Moderately Useful*). Data recording (52.3%) and customizable avatars (50%) were rated as the least useful VR features.

### 3.5 Other tele-VR simulations and features

After responding to the tele-VR simulation and feature questions, participants were given the option to suggest other tele-VR simulations and features that might be useful for therapy with their telehealth clients. We received optional responses from 73 participants. After excluding 6 null responses such as “not that I can think of,” we segmented responses with multiple features or simulations into discrete suggestions (*n* = 72). We then grouped discrete simulation suggestions into themes of treatment context (Table 3). No discernable themes emerged among the features requested (*n* = 7).

Participants most frequently requested tele-VR simulations for behavioral skills training (*n* = 13, 16.7%). Specific behavioral skills training topics included vocational skills (e.g., job interviews, computer skills, professional communication), independent living skills (e.g., cleaning, living with disabilities, self-care), and social skills (e.g., making phone calls, interacting with peers). Tele-VR simulations for exposure therapy (e.g., hospital rooms, being approached from behind, addiction exposures) were also suggested frequently (*n* = 11%, 13.7%). Other suggestions included simulations for relationship therapies (e.g., parenting, role play), OCD (e.g., compulsive hoarding, contamination), relaxation (e.g., meditation, virtual pets), social situations (e.g., dating, meeting new people, loneliness among elderly), trauma (e.g., grief, homelessness), gender identity or sex (e.g., coming out, presenting as another gender before gender-affirming surgery), sensory stimulation (e.g., the sight, smell, and feel of touching mushy, old rice), play therapy, or dissociative identity disorder. Therapists requested features for conducting psychological and neuropsychological assessments, general psychotherapy, group meetings, support groups, grounding techniques, imagery, and considerations for clients of different ages. One therapist responded with general disapproval of tele-VR:

No, we are sociable mammals—I won’t ever use this technology in my practice. If someone wants to do VR they can come into the office and see me in person. Also exposure therapy has a high attrition rate, more so than [eye movement desensitization and reprocessing]. And in spite of the wealth of the U.S., the people here are still generally unhappy. VR will not change that.

### 3.6 Tele-VR factors

**FIGURE 4:**
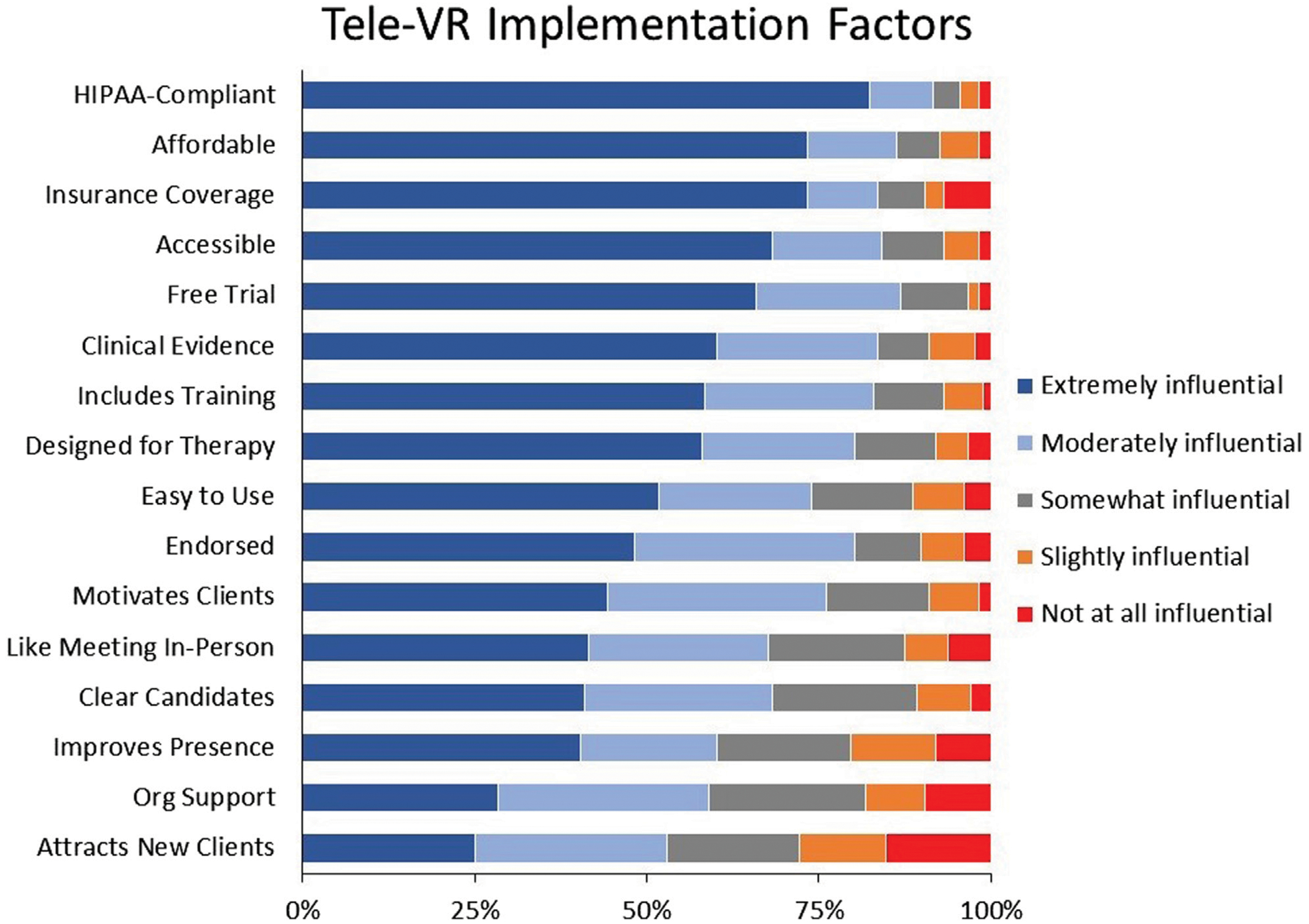
Therapist ratings of tele-VR implementation factors.

Participants rated 16 tele-VR implementation factors on a 5-point Likert scale from Not At All Influential to Extremely Influential (Figure 4). The most influential factor was Health Insurance Portability and Accountability Act (HIPAA) compliance (91.5% rated as Moderately or Extremely Influential), followed by a free trial period (86.9%), affordable adoption (86.4%), accessibility to clients (84.1%), and coverage by health insurance (83.5%). The least influential factors were therapeutic presence (60.2%), organizational support (59.1%), and attracting new clients (52.8%).

## 4 Discussion

Our goal was to explore telemental health therapists’ experiences with and perspectives on telehealth-based VR therapy. The 176 participating therapists reported a variety of mental health specialties and years of service, but most started using telehealth in 2020 and treated adult clients for anxiety, depression, or trauma. The majority of therapists reported no prior experience with VR, having never used VR in their services, and neutral impressions of VR for therapy. Despite this general inexperience with VR, more than half of therapists rated each tele-VR simulation and therapy feature as moderately to extremely useful for their telehealth practices, with the exceptions of simulations for serious accidents, domestic violence, sexual assault, and combat. Therapists’ highest rated tele-VR simulations were for social situations and flying, and their favored features were personalized virtual spaces, tele-VR exercises for clients to complete on their own, and immersive activities. Therapists emphasized practical implementation factors related to revenue and feasibility (i.e., HIPAA compliance, free trial period, insurance coverage, affordability, and accessibility). These findings have practical implications that can inform current and future adoption of tele-VR solutions.

We found that our sample of United States mental healthcare providers had mostly neutral or positive perspectives about VR therapy. In contrast, Australian mental healthcare providers surveyed in 2019 were more positive (65% compared to our 31.2%), less neutral (36% compared to our 56.8%), and less negative (0% compared to our 11.9%) (Chung et al., 2022). While it is not known why perspectives differed across the two samples, it is clear that mental health providers have concerns about adopting VR in their telehealth services. However, 100% of mental health providers who used VR therapy endorsed and recommended it to other providers (Vincent et al., 2021), and clients’ negative reactions to VR therapy remain rare, mild, and temporary in the published literature (Lundin et al., 2023). Larger and more representative sampling will be necessary to better understand therapist perspectives on tele-VR. Only about 6% of therapists in our sample reported using VR in their therapy, compared to 10% of Veterans Affairs care providers and 13.5% of cognitive behavior therapists in previous research (Lindner et al., 2019a; Preston et al., 2022). Future research should examine which mental health providers are using VR for therapy, why therapists may feel skeptical about VR therapy, and evidence-based guidelines to identify when VR therapy would be appropriate, safe, and effective (Rizzo et al., 2023).

It is important to reflect on how therapists in the current study rated the usefulness of tele-VR simulations and features. Therapists’ most favored tele-VR simulations were for social situations and phobias (i.e., flying, heights, enclosed spaces, driving, then animals). This usefulness hierarchy aligns with the landscape of simulations demonstrated in previous VR therapy research (Arnfred et al., 2023). This information can help place mental healthcare as a primary use case of emerging immersive technologies. Declaring therapists’ expectations for clinical VR content may stimulate competition for VR software content offerings, facilitating adoption and growth for clinical VR. However, these therapist ratings may also signal misalignments in perceptions. For example, therapists in the current study rated social situations as their most useful tele-VR simulation but VR avatars as the least useful tele-VR feature. There is growing evidence that personalizing one’s VR avatar can foster social presence, immersion, and embodiment, which then positively impact VR therapy experiences (Aymerich-Franch et al., 2014; Gall et al., 2021; Matamala-Gomez et al., 2021). This potential disconnect between therapists’ high perceived usefulness of social simulations and low perceived usefulness of VR avatars may be a result of the current sample’s limited experience with VR. It would be valuable for future research to investigate tele-VR perceptions, preferences, and experiences among expert VR therapists.

Therapists indicated that the least useful tele-VR simulations were related to trauma (i.e., serious accidents, domestic violence, sexual assault, and combat). While at least 39% of therapists rated these simulations as useful, this finding is remarkable since VR-based exposure therapy (VRET) for trauma has been one of the most widespread and successful clinical applications of VR (Carl et al., 2019; Deng et al., 2019; Kothgassner et al., 2019; Rizzo et al., 2023). It may be the case that therapists in this study were especially skeptical due to the proposed combination of VR therapy, telehealth, and exposure therapy. Participants’ unfamiliarity with VR therapy may have stacked negatively with existing telehealth adoption pains and the notorious difficulty of providing exposure therapy for trauma (Lindner et al., 2019a; Cowan et al., 2019; Pittig et al., 2019). For example, therapists unfamiliar with VR may doubt its reliability, especially if they have experienced unstable internet connection in their telehealth sessions, which may make the combination of VR and telehealth an unacceptable risk for clients undergoing treatment for severe trauma. Nevertheless, tele-VR for the treatment of trauma disorders represents a promising opportunity to expand access and flexibility of care (Morland et al., 2020; Schiavone et al., 2021). More research is needed to understand therapists’ reservations about tele-VR for trauma and its performance in clinical settings.

The five most influential tele-VR implementation factors were HIPAA compliance, a free trial period, insurance coverage of tele-VR services, affordable adoption, and accessibility to patients. Most of these implementation concerns can be reduced if therapists could try tele-VR before having to make substantial investments in equipment, software, or training. The importance of insurance coverage cannot be understated as 3 out of 4 therapists in this study reported private and public health insurance were their primary sources of reimbursement. Relatedly, insurance policies may not cover a service if its delivery is not HIPAA-compliant, just as patients are unlikely to embrace VR if their privacy and security are uncertain (AMA, 2022). At the time of this writing (April 2024), Meta VR devices are not explicitly HIPAA compliant while Pico VR devices (Meta’s closest competitor) may be banned in the United States due to affiliation with a potentially competing government (Fields, 2023). Despite limited offerings from hardware manufacturers, the United States Centers for Medicare and Medicaid Services created a billing code for “virtual reality cognitive behavioral therapy devices” effective 1 April 2023 (Murphy, 2023). It will be essential to advocate for these five critical factors–HIPAA compliance, free trial periods, insurance coverage, affordability, and accessibility–to establish healthcare as a primary market for VR hardware and software technologies.

Therapists in the present study rated enhanced therapeutic presence and attracting new clients to be some of the least influential tele-VR implementation factors. Therapists’ lower prioritization of using tele-VR to attract new clients may be an artifact of already unsustainable case loads (Zangani et al., 2022). However, the lower prioritization of enhanced therapeutic presence may be important to investigate further. Immersion (feeling engaged in a simulation), embodiment (feeling that one is inhabiting a simulated body), and presence (feeling that one is inhabiting a simulated place) are some of the uniquely additive benefits of VR therapy (Hilty et al., 2020; Lindner, 2021). If therapists do not view these key features of VR to be compelling for telemental healthcare, the paths to adoption and implementation may be challenging. Future research should investigate why therapists may feel immersion is not a majorly influential prospect of tele-VR, as well as explorations between potentially related constructs like presence in therapeutic alliance and presence in immersive experiences (Slater et al., 2022; Aafjes-Van Doorn et al., 2023; Chard et al., 2023).

## 5 Limitations and future research

These results should be interpreted with several limitations in mind. We recruited participants from a single telehealth platform who may not be representative of all United States mental health professionals. While the demographics of these participating therapists aligned with those of the overall United States psychology workforce (American Psychological Association, 2022), mental healthcare is a diverse and growing field that future research should aim to capture more representatively.

Most therapists had no experience with VR and almost none had used VR clinically. This means therapist ratings in the current study were mostly hypothetical responses to our brief video rather than direct clinical experience with the myriad of tele-VR simulations, features, and implementation factors presented in the survey. Therapists likely had differing prior knowledge about VR which, combined with our sampling procedures, may have masked meaningful differences in personal or personal or professional demographics. Sampling of VR therapists has been a challenge in research. For example, researchers in 2020 conducted an extensive search and found only 128 practicing VR therapists in the United States, of whom only 17 completed the study (Vincent et al., 2021). It will be vital for researchers to collaborate with experienced VR therapists to understand the real-world experiences, opportunities, and risks of VR for mental healthcare.

We presented a limited selection of tele-VR simulations, features, and factors based on previous research. Though we provided participants the opportunity to suggest other important options for tele-VR, there remains a growing variety of VR simulations, features, and factors that may enable tailored therapy experiences. Examples include 360-degree video cameras (Ionescu et al., 2021; Kupczik et al., 2022; Best et al., 2023), entirely self-paced VR interventions (Shin et al., 2021; Kahlon et al., 2023), customizable VR avatars and embodied interactions (Zhang Brandstätter et al., 2023; Davis and Alexanian, 2024), techniques to evoke sensory illusions in VR related to therapy (Piitulainen et al., 2022; Krell and Wettmann, 2023), and the increasing viability of augmented and mixed reality (Zhang Z. et al., 2023; Hasan et al., 2023). It is important to explore with caution and transparency, especially in light of growing risks in online identity, cybersecurity, and potential abuses of automation technologies (Inkster et al., 2023; Rudschies and Schneider, 2024).

We surveyed mental health providers but not mental health clients. In previous research. Clients who received therapy for anxiety perceived VRET to be significantly more interesting, comforting, and effective than *in-vivo* exposure (Levy et al., 2023). However, it is not yet known how clients perceive VRET delivered over telemedicine. Interestingly, a recent study of in-person VRET found that clients valued the physical presence of their therapist much more than the therapists (Mayer et al., 2022). Future studies should investigate not only client perspectives on tele-VR, but also where client perspectives diverge from those of their therapists, and how those differences may affect care and outcomes.

## 6 Conclusion

We found that half of telemental health providers were inexperienced with VR, had never used VR clinically, and had neutral perspectives of VR therapy. However, they were interested by the prospect of telehealth-based VR therapy and rated various simulations, features, and factors as useful and influential to their adoption of tele-VR, regardless of their individual demographics or practice characteristics. Additional tele-VR suggestions included modules for evidence based practices such as behavioral skills training and meditation, as well as feature requests like immersive assessments and therapeutic content for clients to complete on their own. HIPAA compliance, insurance reimbursement, affordability, and accessibility were the most influential implementation factors. These results extend the findings of previous research on therapists’ experiences and perceptions of VR, and can inform the design of current VR and telehealth solutions for scalable mental healthcare.

## Supplementary Material

Survey Items

## Figures and Tables

**TABLE 1:** Participant demographics.

Variable	*M* (*SD*), range, mode

Age	50.3 (12.5), 25–79, 52

Years practicing mental health	18.9 (9.8), 2–17, 10

Years using telehealth	3.8 (1.6), 1–10, 3

	*N* (%)

**Gender**	
Female	133 (75.57%)
Male	41 (23.30%)
Other	2 (1.14%)

**Ethnicity**	
Non-Hispanic	164 (93.18%)
Hispanic	12 (6.82%)

**Race**	
Multiracial	7 (3.98%)
Asian	10 (5.68%)
Black	11 (6.25%)
White	148 (84.09%)
American Indian	0 (0%)
Pacific Islander	0 (0%)

**Degree**	
Master’s	99 (56.25%)
PhD	77 (43.75%)

**Specialty**	
Marriage and family therapy	30 (17.05%)
Social work	31 (17.61%)
Mental health counselor	48 (27.27%)
Psychologist	67 (38.07%)
Behavior analyst	0 (0%)
Psychiatrist	0 (0%)
Psychiatric nurse	0 (0%)

**Practice organization**	
Individual provider	128 (72.73%)
Small clinic	42 (23.86%)
Large clinic	6 (3.41%)
Educational institution	0 (0%)

**Primary reimbursement**	
Public insurance	15 (8.52%)
Client out-of-pocket	47 (26.71%)
Private insurance	114 (64.77%)

**Clients treated via telehealth**	
Few (1%–24%)	5 (2.84%)
Some (25%–49%)	30 (17.05%)
Most (50%–74%)	34 (19.32%)
Almost all (75%–99%)	36 (20.46%)
All (100%)	71 (40.34%)

**Primary client age group**	
Children (0–10 years)	3 (1.71%)
Adolescents (11–17)	4 (2.27%)
Adults (18–64)	148 (84.09%)
Older adults (+65)	3 (1.71%)
All ages	18 (10.23%)

**Primary disorders treated** *	
Anxiety	139 (87.42%)
Depressive	127 (79.87%)
Trauma	101 (63.52%)
Bipolar	15 (9.43%)
Neurodevelopmental	14 (8.81%)
Addictive	10 (6.29%)
Other	9 (5.66%)
Obsessive-compulsive	9 (5.66%)
Gender	6 (3.77%)
Personality	6 (3.77%)
Eating	5 (3.14%)
Sexual	4 (2.52%)
Dissociative	3 (1.89%)
Psychotic	2 (1.26%)
Sleep	2 (1.26%)
Somatic	1 (0.63%)
Neurocognitive	1 (0.63%)
Paraphilic	1 (0.63%)
Elimination	0 (0%)

* = responses not mutually exclusive.

**TABLE 2:** VR background.

Variable	*n* (%)

**General VR experience**	
No experience	90 (51.14%)
Slightly experienced	48 (27.27%)
Somewhat experienced	18 (10.23%)
Moderately experienced	12 (6.82%)
Extremely experienced	8 (4.55%)

**Use of VR in therapy**	
Never	75 (42.61%)
Once	0 (0%)
Rarely	1 (0.57%)
Sometimes	4 (2.27%)
Frequently	6 (3.41%)

**Overall impression of VR**	
Very negative	6 (3.41%)
Somewhat negative	15 (8.52%)
Neutral	100 (56.82%)
Somewhat positive	37 (21.02%)
Very positive	18 (10.23%)

**TABLE 3:** Other useful tele-VR simulations and features requested.

Simulations	Demonstrative quote	*n (%)*
**Behavioral skills training**		
Simulations for acquiring and practicing specific vocational, independent living, or social skills	“Skill training simulations (job interviews, ordering in restaurants, setting up a bank account, etc.)”	13 (18.1%)
**Exposure therapy**		
Objects or situations related to phobia, anxiety, or addiction	“Triggers to addiction (bar, casino, dealers, paraphernalia, porn, etc.) to practice relapse prevention plan *in vivo*”	11 (15.3%)
**Relationship therapy**		
Arrangements to facilitate remote, group-oriented therapy for couples, families, or other relationships	“Family therapy in which high conflict is present.”	8 (11.1%)
**Obsessive compulsive disorder**		
Simulations related to compulsive behaviors or thoughts about harm, contamination, or hoarding	“OCD scenarios, phone recording conversations, being alone in the woods, dying in different scenarios, injuring/harming people scenarios”	7 (9.7%)
**Mindfulness and Relaxation**		
Interactive environments to facilitate states of calm or safety	“Participating in a outdoor activity, meditation, interacting with nature (calming), mindfulness, bright light therapy”	6 (8.3%)
**Social anxiety**		
Environments in which the client can interact with real or simulated others	“Social interactions with new people”	6 (8.3%)
**Trauma and grief**		
VR arrangements for therapies related to confronting, processing, or responding to trauma or loss	“Grief and loss? Pain from losing a loved one or pet?”	5 (6.9%)
**Gender or sexuality**		
Embodiment of VR avatars to simulate living as an individual of another sex, gender, or sexuality	“VR tech might be helpful in allowing someone to see themself as if they were post-transition in order to explore gender identity, or for addressing sexual performance issues in a lower-stakes environment”	4 (5.6%)
**Sensory**		
Using VR features to recreate the experience of specific perceptions or sensations	“VR seems to provide the visual, kinesthetic aspects of situations …. but in trauma there are other triggers such as smells (olfactory), touch, etc. Not sure how VR would include these.”	2 (2.8%)
**Play therapy**		
VR treatment modules for roleplay, art therapy, and play therapy	“Embodiment work, physically expressive arts like dance or movement”	2 (2.8%)
**Dissociative identity disorder**		
Immersive simulations of auditory, visual, or other hallucinations	“Internal experience of DID”	1 (1.4%)
**Features**		7 (9.7)
1. “Psychological assessment for intelligence measures, neuropsychological testing, and Rorschach and other projective measures.”		
2. “I wouldn’t need a lot of simulations. Just being able to meet with clients in VR, would be very helpful.”		
3. “Imagery”.		
4. “Consider age range/developmental needs/differences”.		
5. “Virtual support group meetings”.		
6. “Grounding opportunities or ways to calm the nervous system [after exposure].”		
7. “General psychotherapy”.		

## Data Availability

The raw data supporting the conclusion of this article will be made available by the authors, without undue reservation.
