# Supplementary material for: Mental health providers are inexperienced but interested in telehealth-based virtual reality therapy: survey study
Source: Front Virtual Real. Author manuscript; Available in PMC 2024 Sep 3. (PMC11370306; doi:10.3389/frvir.2024.1332874)
Supplement: Survey Items [file NIHMS2017816-supplement-Survey_Items.pdf]

Appendix 1. Survey questions.

Screening

1. What is your age, in years?
2. Are you fluent in English?
    a. Yes
    b. No
3. What is the most advanced degree you've obtained?
    a. I have not obtained a Master's or Doctoral degree
    b. Master's degree
    c. Doctoral degree
4. Which title best describes your profession?
    a. 1 - Mental Health Counselor (e.g., LMHC, LPC)
    b. 2 - Social Worker (e.g., LCSW)
    c. 3 - Marriage and Family Therapist (e.g., LMFT)
    d. 4 - Psychologist (e.g., PhD, PsyD)
    e. 5 - Behavior Analyst (e.g., BCBA)
    f. 6 - Psychiatrist (e.g., MD)
    g. 7 - Psychiatric nurse practitioner (e.g., RN, PMH)
    h. 0 - I am not a mental health therapist
5. What percent of your clients do you see via telehealth?
    a. 0 = None (0%)
    b. 1 = Few (1–24%)
    c. 2 = Some (25–49%)
    d. 3 = Most (50–74%)
    e. 4 = Almost all (75–99%)
    f. 5 = All (100%)

Demographics

6. How would you describe your gender?
    a. Female
    b. Male
    c. Non-binary, transgender, or other gender
7. Are you of Hispanic or Latino origin?
    a. Yes
    b. No
8. How would you describe your race?
    a. American Indian or Alaska Native
    b. Asian
    c. Black or African American
    d. Native Hawaiian or Other Pacific Islander
    e. White
    f. Multiracial
9. How long have you been providing mental health services, in years?
10. How long have you been using telehealth to provide mental health services, in years?

11. How would you describe your clinical practice or organization?
    a. Individual practice
    b. Small clinic or network of providers
    c. Large clinic or hospital
    d. Educational setting
12. What is your primary source of reimbursement for your telehealth services?
    a. Public Insurance (e.g., Medicare, Medicaid)
    b. Private Insurance
    c. Client out-of-pocket
13. Which age group do you provide therapy for most often?
    a. Children (0-10 yrs old)
    b. Adolescents (11-17 yrs old)
    c. Adults (18-64 yrs old)
    d. Older adults (65+ yrs old)
    e. All ages
14. Which mental health disorder is the primary focus of your telehealth services? (Pick up to 3)
    a. Other
    b. Neurodevelopmental disorders (e.g., intellectual disabilities, attention-deficit/hyperactivity)
    c. Psychotic disorders (e.g., schizophrenia, delusional disorder, catatonia)
    d. Bipolar disorders
    e. Depressive disorders
    f. Anxiety disorders (e.g., phobia, social anxiety, panic disorder)
    g. Obsessive-compulsive disorders (e.g., hoarding, body dysmorphia)
    h. Trauma and stressor disorders (e.g., PTSD)
    i. Dissociative disorders
    j. Somatic disorders
    k. Feeding or eating disorders (e.g., anorexia, bulimia, pica)
    l. Elimination disorders (e.g., enuresis, encopresis)
    m. Sleep-wake disorders (e.g., insomnia, narcolepsy, nightmares)
    n. Sexual dysfunctions
    o. Gender dysphoria
    p. Substance abuse and addictive disorders
    q. Neurocognitive disorders (e.g., Alzheimer's, TBI, Parkinson's)
    r. Personality disorders
    s. Paraphilic disorders
15. (Optional) Please describe the primary focus of your telemental health services.

VR Experience

16. How would you describe your experience with Virtual Reality?
    a. No experience
    b. Slightly experienced
    c. Somewhat experienced
    d. Moderately experienced

e. Extremely experienced

17. How often have you used Virtual Reality to provide mental health therapy?
    a. Never
    b. Once
    c. Rarely
    d. Sometimes
    e. Frequently
18. Based on your current knowledge, what is your overall impression of Virtual Reality for mental health therapy?
    a. Very negative
    b. Somewhat negative
    c. Neutral
    d. Somewhat positive
    e. Very positive

Tele-VR Simulations

19. If you were considering Virtual Reality for your telehealth clients, how useful might these Virtual Reality simulations be? (Not useful at all 1 - 5 extremely useful)
    a. Driving a car
    b. Being in an airplane
    c. Being up high (e.g., top floor of a skyscraper)
    d. People, crowds, or social situations (e.g., school, public speaking, parties)
    e. Errands outside the home (e.g., grocery shopping, doctor visit, going to the bank)
    f. Enclosed spaces (e.g., closet, elevator, MRI machine)
    g. Small animals (e.g., dogs, spiders, snakes)
    h. Medical procedures (e.g., blood draw, surgery, dental work)
    i. Combat (e.g., military)
    j. Domestic violence
    k. Sexual assault
    l. Serious accidents (e.g., motor vehicle collision, major injury, natural disasters)

Tele-VR Features

20. If you were considering Virtual Reality for your telehealth clients, how useful might these Virtual Reality features be? (Not useful at all 1 - 5 Extremely useful)
    a. Personalizing therapeutic spaces (e.g., co-constructing a client's "safe place").
    b. Therapists and clients can customize how they look and dress in Virtual Reality (i.e., avatars).
    c. Immersive forms of interaction like games, drawing, or constructing scenes in Virtual Reality.
    d. Sharing internet content together in Virtual Reality (e.g., viewing websites, images, or videos).
    e. Ways to record session notes and collect client data in Virtual Reality .
    f. Virtual Reality mental health exercises for clients to complete on their own (i.e., for homework).
21. (Optional) Are there other Virtual Reality features or simulations that would be useful for

your telehealth therapy? Please describe.

Tele-VR Factors

22. If you were considering Virtual Reality for your telehealth clients, how influential might these factors be? (Not at all influential 1 - 5 Extremely influential)
    a. Virtual Reality is accessible to my telehealth clients regardless of their age, sex, race, or other socioeconomic factors.
    b. Virtual Reality helps my telehealth clients stay motivated during treatment.
    c. Virtual Reality helps my telehealth clients feel that I am more present in their therapy experience.
    d. There is strong clinical evidence supporting Virtual Reality for telemental health care.
    e. Virtual Reality makes it easier to do therapy "in the field" with my telehealth clients, as if we were meeting in-person (e.g., at a shopping mall, in their home, at their school).
    f. It is clear which clients are good candidates for Virtual Reality-based telemental health care.
    g. Virtual Reality is easy to use for therapy with my telehealth clients.
    h. The Virtual Reality software was designed specifically for telemental health care.
    i. The Virtual Reality includes training and support (e.g., video tutorials, FAQ, live tech support).
    j. Virtual Reality therapy is secure, private, and in compliance with policies such as HIPAA or GDPR.
    k. Virtual Reality therapy is supported by organizations such as the American Psychological Association and the World Health Organization.
    l. My team members (e.g., admin, staff, IT) and I support Virtual Reality for telemental health care.
    m. Virtual Reality attracts new clients to my telemental health practice.
    n. Virtual Reality for telemental health care is covered by my clients' health insurance.
    o. Virtual Reality is affordable to adopt into my telemental health practice.
    p. I can evaluate Virtual Reality in my telemental health practice before purchasing (e.g., trial period).
